# Comparison of hematologic parameters, serum electrolytes, and lipid profiles among dyspeptic patients with and without *Helicobacter pylori* infection attending Jimma Medical Center, Jimma, South West Ethiopia

**DOI:** 10.1371/journal.pone.0310047

**Published:** 2024-11-04

**Authors:** Negussie Sarbecha, Minale Fikade, Tesaka Wondimnew, Kumsa Kene, Negawo Kebede, Habtemariam Gebresillasie, Zerihun Assefa

**Affiliations:** 1 Department of Biomedical Sciences, School of Medicine, Madda Walabu University, Robe, Ethiopia; 2 Department of Biochemistry, School of Biomedical Sciences, Jimma University, Jimma, Ethiopia; Wollo University, ETHIOPIA

## Abstract

**Background:**

About half of the world’s populations are infected with *Helicobacter pylori*, which may create atherogenic lipid profiles and contribute to atherosclerosis and related cardiovascular disease. Furthermore, it has been connected to hematological symptoms like anemia. Even though the high prevalence of *H*.*pylori* and its associated complications, including cardiovascular disease and anemia, in Ethiopia, there is little data regarding the risk factors associated with *H*.*pylori* infection, such as hematologic parameters, electrolyte imbalances, and lipid profiles.

**Methods:**

A comparative cross-sectional study design with a consecutive sampling technique was employed at Jimma Medical Center among 108 dyspeptic patients. Five (5) ml of blood samples were collected from each participant, and serum was extracted and analyzed using a COBAS 6000 core for the lipid and electrolyte, and whole blood was used with a SYSMEX XN 550 to determine hematological parameters. Data were entered in to Epi-data version 4.6 and exported to SPSS version 25 for analysis. Simple descriptive statistics and chi-square test were used to present the socio-demographic characteristics of the study subjects. Student t-test was used for data comparison and p-value < 0.05 was considered statistically significant.

**Results:**

*H*.*pylori* infected patients had significantly decreased levels of red blood cell count (p = 0.002), hemoglobin (p = 0.012), mean corpuscular hemoglobin concentration (p < 0.001), platelet count (p = 0.001), and sodium level (p = 0.006) when compared to the uninfected group. However, total cholesterol (p = 0.001), and low density lipoprotein (p = 0.021) were increased in *H*.*pylori* infected patients when compared to the uninfected group.

**Conclusions:**

This study revealed that *H*.*pylori* infection can cause derangements of hematologic parameters, electrolyte imbalances, and alterations of lipid parameters which considered as risk factors for anemia and cardiovascular diseases.

## Introduction

Dyspepsia is typically used to describe upper abdominal discomfort that is believed to be caused by the upper gastrointestinal tract and may include symptoms such as abdominal bloating, belching, epigastric pain, heartburn, acid regurgitation, early satiety, nausea, and a sense of slow or abnormal digestion [1, 2]. The main causes of dyspepsia are functional dyspepsia (70%), peptic ulcer disease (PUD) (10%), gastroesophageal reflux disease (GERD) (5%), and gastric cancer (1%) [3]. Functional dyspepsia is caused by *H*.*pylori* infection, delayed gastric emptying, impaired stomach accommodation, diet, lifestyle factors like chewing khat, drinking alcohol, and smoking, as well as psychosocial factors like depression, stress, and anxiety [4].

*H*.*pylori* is a spiral-shaped gram-negative commensal bacterium that inhabits the human stomach [5]. For almost 40 years, *H*.*pylori* has been recognized as a serious and widespread human bacterial pathogen [6]. Factors like socioeconomic status, poverty, crowded living conditions, sanitation, ethnicity, and unsafe water and food can contribute to the incidence of *H*.*pylori* infection [7]. Infection rates vary by region, but over the past three decades, the number of infected people has maintained or even increased due to population expansion, reinfection, and recrudescence due to unsuccessful eradication [8].

It was estimated that 4.4 billion people worldwide tested positive for *H*.*pylori* in 2015, with Asia (54.7%), the Caribbean and Latin America (63.4%), and Africa (79.1%) having the greatest prevalence rates [9]. In Ethiopia, the overall polled prevalence of *H*.*pylori* infection was 52.2% [10].

*H*.*pylori* has been associated with numerous diseases both inside and outside of the stomach area because of the very virulent substances it produces [11]. Around 80% of duodenal ulcers and 50% of gastric ulcers are connected with this infection [12]. *H*.*pylori* is one of the infections designated by the World Health Organization (WHO) as a carcinogen and the main risk factor for non-cardia gastric adenocarcinoma [13, 14].

The settlement of the stomach by *H*.*pylori* initiates the surrounding area’s inflammation by attracting lymphocytes and neutrophils [15, 16]. Infection and inflammation induces the activation of different cytokine like IL-1, IL-6, and TNF, which trigger the alteration of lipid metabolism [17]. The activation of cytokines can change lipid metabolism through the initiation of hepatic fatty acid synthesis, stimulation of adipose tissue lipoprotein lipase, and increased lipolysis [18]. People with *H*.*pylori* infection have atherogenic lipid profiles, which could induce atherosclerosis and related cardiovascular diseases like peripheral vascular diseases, stroke, and myocardial infarction [19, 20]. Cardiovascular diseases are the leading cause of disability and are accountable for around thirty-five million deaths per year, of which 85% are in low socioeconomic countries [21].

Furthermore, *H*.*pylori* has been linked to hematological symptoms like anemia [22]. Approximately 1.62 billion individuals are affected by anemia worldwide, and from those, almost half of all the incidence of anemia is caused by iron deficiency [23]. The incidence of iron deficiency anemia increased by 2.6-fold in *H*.*pylori* infection patients [24]. The association between *H*.*pylori* and iron deficiency may be due to bleeding and occult bleeding loss secondary to PUD, gastric malignancy, and chronic erosive gastritis that occur due to surface virulent factors of *H*.*pylori* like Cag A or due to iron uptake and utilization by *H*.*pylori* and may be due to decreased absorption of iron and intrinsic factor deficiency secondary to chronic gastritis and hypochlorhydria [25]. A lack of intrinsic factors results in less vitamin B12 being absorbed, which could change hematologic parameters [26].

*H*.*pylori* is also accountable for a decrease in platelet count because of the molecular mimicry with *H*.*pylori* CagA, which causes the generation of autoantibodies against *H*.*pylori* CagA and the cross-reactivity of these antibodies with platelet surface antigens [27]. *H*.*pylori* attachment to gastric epithelial cells enhanced Na-K-ATPase ubiquitylation and decreased its surface and total levels [28]. Alteration of Na + K + ATPase might affect the level of serum sodium levels by inhabiting the efflux of sodium out of the cell. *H*.*pylori* infection has also had a significant association with chronic kidney disease, which may have been related to electrolyte derangement [29, 30]. Any changes in serum electrolyte levels increase the risk of cardiovascular morbidity and its associated death [31].

Regarding the association between the presence of *H*.*pylori* infection and hematologic parameters, electrolyte imbalance, and lipid profiles, the findings of several researchers have been inconsistent and occasionally contradictory [23, 32–34]. Although *H*.*pylori* infection is very common in Ethiopia [10], there is little data available on the association between *H*.*pylori* infection and hematological parameters and lipid profiles. As far as our knowledge, there is no study on the assessment of electrolyte imbalance among *H*.*pylori* dyspeptic patients in Ethiopia. Therefore, this study was conducted to fill the gap in earlier findings and to look into the relationships existing in our nation that could aid in the inpatient care and complication prevention of dyspeptic patients who are *H*.*pylori* positive.

## Materials and methods

### Study design, period and area

An institutional-based comparative cross-sectional study was conducted from February 5 to April 20, 2023, at Jimma Medical Center (JMC), Ethiopia. Geographically, it is situated in Jimma town, which is 352 kilometers southwest of Addis Ababa, the capital city of Ethiopia. JMC is one of the oldest hospitals in Ethiopia, with over 800 beds and a capacity for 16,000 inpatients, 220,000 outpatient attendants, and 12,000 emergency cases. It serves a catchment population of about 15 million people and is the only teaching and referral facility in southwest Ethiopia.

### Study participants

The study populations for this study were all dyspeptic patients attending JMC during the study period. The study was conducted on two groups of the study population: one group of dyspeptic patients with *H*.*pylori* positive and the other group of dyspeptic patients without *H*.*pylori* infection. Subjects with an age <18 years, a history of *H*.*pylori* eradication therapy within the past four weeks, on lipid-lowering medications, with known hematological disorders, pregnant women, renal disease, liver disease, and diabetes were excluded from the study.

### Sample size determination and sampling technique

The sample size was determined by using the formula for the comparison of two means with equal sample sizes in each group by considering the following assumptions: a confidence interval of 95%, a power of 80%, the mean and standard deviation of Hgb from the previous study [35], 13.32 and 1.56 for the *H*.*pylori*-infected group, and 14.25 and 1.89 for the *H*.*pylori* negative group. This gives a total of 108 study participants, with 54 in each group. A consecutive sampling technique was employed to recruit the required sample size.

### Data and sample collection and laboratory methods

After the study participants were asked for their consent, approximately 3 grams of stool were collected in a sterile, single-use bottle and analyzed by stool antigen test kits from Wondfo (Guangzhou Wondfo Biotech Co., China.) to detect *H*.*pylori* antigen. Data on sociodemographic characteristics were collected using a structured questionnaire by trained nurses. In addition, 5 ml of venous blood was withdrawn from the study participants. Two-point-five (2.5) ml of blood sample was used to measure serum electrolytes (Na+, K+, and Cl-) and lipid profiles (TC, HDL-cholesterol, LDL-cholesterol, and triglycerides) using the COBAS 6000 core (Hitachi High-Technologies Corporation, Japan), while 2.5 ml of blood sample was used to measure hematological parameters (Hgb, HCT, RBC, MCV, MCH, MCHC, WBC, and PLT) using SYSMEX XN 550 per the standard operating procedure (SOP).

### Data processing and analysis

The data were coded, entered into EpiData version 4.6, and exported to the SPSS version 25 package, where the different variables were tested and analyzed. The Kolmogorov-Shapiro test was used to determine whether the distribution of the data was normal. Simple descriptive statistics and the Chi-square test were used to present the socio-demographic characteristics of the study subjects, and outcomes variables were presented as mean ± standard deviation and compared using two independent t-tests for normally distributed data and media ± interquartile range and compared using the Mann-Whitney U-test for not normally distributed data to test for significant differences between groups, and a P value < 0.05 was considered significant.

### Ethics approval and consent to participation

After ethical approval, ethical clearance was obtained from the institutional review board of Jimma University Institute of Health with a reference number (JUIH/IRB2/2/22). Written informed consent was obtained from all the study participants. All patients with abnormal findings were linked to the physician, and the information was maintained confidentially.

## Results

### Sociodemographic characteristics of the patients

This study included a total of 108 dyspeptic patients, with 54 *H*.*pylori*-positive patients and 54 *H*.*pylori*-negative patients. The mean age of *H*.*pylori*-negative patients was 31.57 with a standard deviation of ±11.46 and 33.15 with a standard deviation of ±10.55 years for *H*.*pylori-*infected patients. Among the study subjects, 57 (52.78%) were women, and 56 (51.9%) of the study participants were rural residents. All sociodemographic characteristics were not significantly associated with *H*.*pylori* infection (Table 1).

**Table 1:** Sociodemographic characteristics of study participants (N = 108, JMC, Jimma, 2023) (Pearson’s Chi-square test).

Variables	Categories	*H*. *pylori-*Infected Group	*H*. *pylori*-negative Group	Chi-square
Gender	Male	26(48.15%)	25(46.30%)	0.85
	Female	28(51.85%)	29(53.70%)	
Age in year	18–27	23(42.59%)	23(42.59%)	0.29
	28–37	14(25.93%)	19(35.19%)	
	38–48	13(24.07%)	6(11.11%)	
	≥48	4(7.41%)	6(11.11%)	
Educational status	Illiterate	18(33.33%)	12(22.22%)	0.14
	Primary school	13(24.07%)	7(12.96%)	
	Secondary school	14(25.93%)	20(37.04%)	
	College/university	9(16.67%)	15(27.78%)	
Marital status	Single	7(12.96%)	16(29.63%)	0.63
	Married	43(79.63%)	32(29.26%)	
	Divorced	4(7.41%)	6(11.11%)	
Residence	Urban	23(42.59%)	29(53.70%)	0.25
	Rural	31(57.41%)	25(46.30%)	

**Abbreviations:** *H*.*pylori*, *Helicobacter pylori*

### Comparisons of hematologic parameters in study participants

The mean (SD) of hematological parameters of patients diagnosed with *H*.*pylori* and those without *H*.*pylori* infection were compared. Accordingly, red blood cell mean level (RBC = 4.66 ± 0.52 × 106/μL vs. 4.99 ± 0.6 × 106/μL), hemoglobin (Hgb = 13.62 ± 1.69 g/dL vs. 14.48 ± 1.81 g/dL), mean corpuscular hemoglobin concentration (MCHC = 31.79 ± 2.03 g/dl vs. 33.46 ± 1.7 g/dl), and platelet count (PLT = 260.02 ± 70.14 x103/μL vs. 288.85 ± 49.86 x103/μL) were all significantly lower in the *H*.*pylori-*infected than the *H*.*pylori*-negative group, with p-values of 0.002, 0.012, <0.001, and 0.001, respectively. The mean level of white blood cell counts (WBCs) was slightly higher among participants who had positive *H*.*pylori* compared to the mean participants with negative results. However, there is no statistically significant difference (P > 0.05) (Table 2).

**Table 2:** Comparisons of mean values of hematologic parameters of *H*. *pylori*-infected patients and non-infected study participants at JMC, 2023.

Hematologic parameters	*H*. *pylori*-positive Group Mean ±SD	*H*. *pylori*-negative Group Mean ±SD	p-value of t-test
RBCx10^6^/μL	4.66±0.52	4.99±0.60	0.002
Hgb (g/dl)	13.62±1.69	14.48±1.81	0.012
HCT (%)	42.11±4.00	43.61±4.43	0.067
MCV(fl)	85.22±4.43	86.42±4.16	0.147
MCH (pg)	28.57±1.30	28.86±1.46	0.283
MCHC(g/dl)	31.79±2.03	33.46±1.70	< 0.001*
PLT count x10^3^/μL	260.02±70.14	288.85±49.86	0.001*
WBC x10^3^/μL	7.94±2.75	7.29±2.38	0.189

**Note:** P-value < 0.05 is considered statistically significant. * P-value of the Mann-Whitney U-Test, which is expressed by the median ± interquartile range

**Abbreviations:** μL, microliters; fl femtoliters; g/dl, gram per deciliter; Hgb, hemoglobin; *H*. *pylori*, *Helicobacter pylori*; RBC, red blood cell; HCT, hematocrit; MCV, mean cell volume; MCH, mean cell hemoglobin; MCHC, mean cell hemoglobin concentration; %, percentage; pg, pictogram; PLT, platelet; WBC, white blood cell; SD, standard deviation.

### Comparisons of serum electrolyte level in study participants

The serum median level of sodium (Na^+^) was significantly lower in the *H*.*pylori-*infected group when compared to the *H*.*pylori-*negative group (p = 0.006). However, the median serum levels of potassium (K+) and chlorine (Cl-) were not statistically significant between the two groups (p > 0.05) (Table 3).

**Table 3:** Comparisons of electrolyte level of *H*. *pylori*-infected and non-infected study participants at JMC, 2023 (Mann–Whitney U-Test).

Electrolytes	*H*. *pylori*-positive Group Median (IQR)	*H*. *pylori*-negative Group Median (IQR)	p-value
Na (mmol/L)	141(13)	143(6)	0.006
K (mmol/L)	6.02(4.05)	5.44(1.9)	0.147
Cl (mmol/L)	106.1(5.5)	107.3(4.4)	0.086

**Note:** P<0.05 = statistically significant value.

**Abbreviations**: Na, Sodium; K, Potassium; Cl, Chlorine; IQR, Interquartile range

### Comparison of lipid profiles among study participants

The mean serum levels of low-density lipoprotein and total cholesterol were significantly higher among *H*.*pylori-*positive patients when compared to *H*.*pylori-*negative patients, with p-values of 0.012 and 0.02, respectively. Even though there was a higher level of triglyceride and lower levels of high-density lipoprotein among the *H*.*pylori*-infected group, there was no significant difference between the two groups (P > 0.05) (Table 4).

**Table 4:** Comparisons of mean values of lipid profiles of *H*. *pylori*-infected and non-infected study participants at Jimma Medical Hospital, 2023.

Lipid Profiles	*H*. *pylori* Status		P-value of t-test
	Positive, Mean (SD)	Negative, Mean (SD)	
HDL-C(mg/dL)	37.96(8.12)	40.03(8.52)	0.074*
LDL-C(mg/dL)	109.89(23.54)	97.54(26.49)	0.012
TG(mg/dL)	140.57(38.76)	129.12(43.74)	0.152
TC(mg/dL)	167.12(39.54)	149.09(39.74)	0.02

**Note:** P<0.05 = statistically significant value. * P-value of Mann–Whitney U-Test

**Abbreviations**: HDL, high-density lipoprotein; LDL, low-density lipoprotein; TC, total cholesterol; TG, triglycerides.

## Discussion

*H*.*pylori* has been known for about 40 years as a serious human pathogen that affects half of the world`s population and continues to be a leading cause of mortality and morbidity globally [6]. It has been associated with numerous diseases both inside and outside of the stomach area because of the very virulent substances it produces [11]. Studies revealed that *H*.*pylori* infection was connected with derangement of some hematologic parameters, electrolyte levels, and lipid profiles, which may be related to different diseases [36–38].

According to the current study, *H*.*pylori-*positive patients had significantly lower mean RBC concentrations than *H*.*pylori-*negative patients (p 0.002). This result was consistent with findings from research carried out in Palestine and Sudan [39, 40]. RBC counts may have decreased in *H*.*pylori*-positive patients as a result of occult blood loss secondary to peptic ulcers or erosion of chronic gastritis [25]. However, the results of this study were in contrast with previous studies [39] that described no significant difference in RBC count between the two groups. This difference may be due to methodology and socio-demographic characteristics.

In addition, our study revealed that the mean level of Hgb was significantly lower among *H*.*pylori*-positive patients when compared to *H*.*pylori*-negative participants. This result was in line with other studies conducted in Egypt and Ethiopia [41, 42]. *H*.*pylori’s* contribution to the decrement of Hgb count might be due to a change in the pH of the gastric mucosa that causes a decreased level of ascorbic acid, which leads to a decrease in iron absorption from the diet; long-term blood loss secondary to PUD, gastric malignancy, and chronic erosive gastritis that occur due to surface virulent factors of *H*.*pylori* like Cag A or due to iron uptake and utilization by *H*.*pylori*; and an increase in the liver’s production of hepcidin, which leads to a decrease in iron absorption by enterocytes and release from macrophages [43, 44]. However, the result was not in line with the studies conducted in Saudi Arabia [32].

Furthermore, in this study, there was a statistically significant median difference in MCHC (P < 0.001) levels between the two groups, indicating the effect of *H*.*pylori* infection on hematological parameters.

In this study, there was a statistically significant median difference in platelet counts (P = 0.001) between the two groups, as there was higher in *H*.*pylori* negative groups (median (IQR)): 288.85 (49.86×10^3^/μl) versus *H*.*pylori* positive groups 260.02 (70.14×10^3^/μl). Our result is in line with a study conducted in Egypt [41]. *H*.*pylori* might contribute to the decrement in platelet count due to the molecular similarity with *H*.*pylori* CagA and the production of autoantibody against *H*.*pylori* CagA and the cross-reactivity of these antibodies with platelet surface antigens (GP Ia/IIa and GP Ib/IX) and platelet clump due to the presence of von Willebrand factor and anti-*H-pylori* IgG [45]. However, our findings contradict those of studies from Saudi Arabia [32] and Palestine [40], which reported there was no significant mean difference in platelet count between the two groups.

In the current study, median values of sodium (Na+) concentration were lower in *H*.*pylori*-infected patients compared to the *H*.*pylori* negative group, which was statistically significant (P = 0.006). This finding is supported by studies conducted in Saudi Arabia [38]. *H*.*pylori* may have contributed to the drop in sodium concentration due to *H*.*pylori’s* attachment to gastric mucosal cells, which decreases Na-K-ATPase through enhanced ubiquitylation of Na-K-ATPase [28]. Alteration of Na + K + ATPase might affect the level of serum sodium levels by inhabiting the efflux of sodium out of the cell. In addition, *H*.*pylori* produces ammonia, leading to the accumulation of excessively hazardous levels of ammonia, which decreases the absorption of sodium due to damage to the digestive tract by ammonia [46].

Persisted *H*.*pylori* infection may affect the lipid profile, which leads to increased levels of total cholesterol, LDL-c, lipoprotein Lp(a), apolipoprotein apo-B, triglyceride concentrations, and decreased levels of HDL-C and apolipoprotein apoA-1 concentrations [18, 47]. The current study also found that patients with *H*.*pylori* infections had significantly higher levels of total cholesterol and LDL-c than did healthy people (p-values of 0.02 and 0.012, respectively). This result is consistent with earlier researches [48–50].

This could be due to the involvement of cytokines in *H*.*pylori* infection, especially IL-6, IL-1, INF-α, and TNF-α, which affect lipid metabolism through stimulation of hepatic fatty acid synthesis, activation of adipose tissue lipoprotein lipase, and influencing lipolysis [18, 47]. Since *H*.*pylori* increases small intestine mucosal permeability, which allows bacterial toxins to reach the liver through the portal vein and cause hepatic tissue damage, lipid levels can also be altered by direct liver dysfunction [51].

A change in lipid profile may also be caused by *H*.*pylori*-related ghrelin and leptin release or by an imbalance in nutritional absorption as a result of digestive system inflammation, which might lead to atherosclerotic lipid profiles and elevated cardiovascular risk [52]. After *H*.*pylori* was effectively eradicated, LDL returned to levels that were nearly *H*.*pylori-*negative, confirming their close relationship [51]. Contrary to the aforementioned discoveries, some studies did not show the association of *H*.*pylori* infection with altered LDL-c and total cholesterol [37, 53, 54]. These controversial study findings may be explained by the varying study methodologies employed, such as different study populations or sample sizes.

## Limitations of the study

This study used a non-probability sampling technique to recruit the study participants, which may affect the external validity of the findings. In our study, stool bacterial load and the strain of *H*.*pylori* were not evaluated; therefore, the effect of *H*.*pylori* virulence factors could not be identified.

## Conclusions

This study revealed that the mean values of platelet, RBC, Hgb, and MCHC count were significantly decreased in *H*.*pylori*-positive participants when compared to *H*.*pylori*-negative patients. In addition, *H*.*pylori* infection alters the serum electrolytes, which were significant in Na^+^. There were also significant increments in LDL-c and total cholesterol in the *H*.*pylori*-infected group, which created an atherogenic lipid. Hence, dyspeptic patients infected by *H*.*pylori* will be at high risk for anemia, atherosclerosis, and associated cardiovascular disease. Therefore, timely diagnosis and eradication of *H*.*pylori* in the infected patients and evaluation of serum electrolytes, hematologic parameters, and lipid profiles for those infected by *H*.*pylori* were recommended.

## List of abbreviations

CagA: Cytotoxin associated gene A
Hct: Hematocrit
Hgb: Haemoglobin
*H.pylori*: *Helicobacter pylori*
HDL-C: High Density Lipoprotein- Cholesterol
LDL-C: Low Density Lipoprotein-Cholesterol
MCHC: Mean Corpuscular Haemoglobin Concentration)
RBC: Red Blood Cell)
TG: Triglyceride
TC: Total Cholesterol
VacA: Vacuolating cytotoxin gene A
WBC: White Blood Cell
